# Decorin (DCN) Downregulation Activates Breast Stromal Fibroblasts and Promotes Their Pro-Carcinogenic Effects through the IL-6/STAT3/AUF1 Signaling

**DOI:** 10.3390/cells13080680

**Published:** 2024-04-14

**Authors:** Wafaa A. Aljagthmi, Manal A. Alasmari, Maha H. Daghestani, Layla A. Al-Kharashi, Falah H. Al-Mohanna, Abdelilah Aboussekhra

**Affiliations:** 1 Department of Molecular Oncology, King Faisal Specialist Hospital and Research Center, Riyadh 11211, Saudi Arabia; 2 Department of Zoology, College of Science, King Saud University, Riyadh 11451, Saudi Arabia; 3 Department of Pharmacology and Toxicology, Faculty of Pharmacy, King Saud University, Riyadh 11495, Saudi Arabia; 4 Department of Comparative Medicine, King Faisal Specialist Hospital and Research Center, Riyadh 11211, Saudi Arabia

**Keywords:** breast cancer, cancer-associated fibroblasts, cancer stem cells, decorin, IL-6/STAT3 pathway

## Abstract

Decorin (DCN), a member of the small leucine-rich proteoglycan gene family, is secreted from stromal fibroblasts with non-cell-autonomous anti-breast-cancer effects. Therefore, in the present study, we sought to elucidate the function of decorin in breast stromal fibroblasts (BSFs). We first showed DCN downregulation in active cancer-associated fibroblasts (CAFs) compared to their adjacent tumor counterpart fibroblasts at both the mRNA and protein levels. Interestingly, breast cancer cells and the recombinant IL-6 protein, both known to activate fibroblasts in vitro, downregulated DCN in BSFs. Moreover, specific DCN knockdown in breast fibroblasts modulated the expression/secretion of several CAF biomarkers and cancer-promoting proteins (α-SMA, FAP- α, SDF-1 and IL-6) and enhanced the invasion/proliferation abilities of these cells through activation of the STAT3/AUF1 signaling. Furthermore, DCN-deficient fibroblasts promoted the epithelial-to-mesenchymal transition and stemness processes in BC cells in a paracrine manner, which increased their resistance to cisplatin. These DCN-deficient fibroblasts also enhanced angiogenesis and orthotopic tumor growth in mice in a paracrine manner. On the other hand, ectopic expression of DCN in CAFs suppressed their active features and their paracrine pro-carcinogenic effects. Together, the present findings indicate that endogenous DCN suppresses the pro-carcinogenic and pro-metastatic effects of breast stromal fibroblasts.

## 1. Introduction

Breast cancer (BC), the leading cause of cancer-related death among women worldwide, represents a true global health challenge [1]. The breast tumor microenvironment (TME) is a major cancer-promoting factor that encourages tumor progression and spread and plays a significant role in the various treatment responses [2]. Cancer-associated fibroblasts (CAFs), which are the most abundant and active cells within the TME, promote tumor progression through the induction of the epithelial-to-mesenchymal transition (EMT) process and stemness in cancer cells through secretion of high levels of several pro-carcinogenic cytokines such as SDF-1, TGF-β1 and IL-6 [3,4]. These CAF-secreted proteins, in addition to VEGF-A and IL-8, promote angiogenesis [5,6]. The expression of most of these pro-carcinogenic factors is under the control of two major transcription regulatory pathways, NF-κB and STAT3 [7]. The RNA-binding protein AUF1 is a downstream effector of STAT3 and is also a regulator of these genes at the mRNA level [8]. AUF1 is upregulated in most CAFs as compared to their adjacent normal counterparts and plays important roles in the activation of breast stromal fibroblasts [8,9]. The TME also includes the extracellular matrix (ECM), an acellular structure with a variety of components that are essential for maintaining tissue homeostasis. During carcinogenesis, ECM proteins assist in tumor onset and progression [10,11,12].

Decorin (DCN) is an extracellular matrix protein expressed in both stromal and epithelial cells [13]. DCN is involved in the cell cycle, autophagy, mitophagy, and wound repair [14,15]. Also, DCN works as an anti-fibrosis agent by interacting with growth factors, such as the transforming growth factor-β (TGF-β) [16]. Additionally, DCN is involved in controlling tumorigenesis and has been proposed as “a guardian from the matrix” [17]. Indeed, DCN was indetectable within the tumor parenchyma of several cancer types [18]. Furthermore, a reduction in DCN levels was linked to poor prognosis [15,18,19]. Mouse embryonic fibroblasts lacking decorin exhibit greater cellular proliferation, migration and invasion than DCN-sufficient murine fibroblasts [20]. On the other hand, DCN upregulation through ectopic expression of the gene reduced the malignant activity of various cancer cell lines to a more benign state [21]. Therefore, this study was designed to explore the functional role of DCN in breast stromal fibroblasts. The present findings reveal that endogenous DCN suppresses the active features and the pro-carcinogenic effects of breast fibroblasts.

## 2. Materials and Methods

### 2.1. Cells, Cell Culture and Reagents

Breast fibroblast cells NBFs (normal breast fibroblasts, developed from tissues obtained from mammoplastic surgeries), CAFs (cancer-associated fibroblasts) and TCFs (tumor counterpart fibroblasts, isolated from adjacent histologically normal breast cells) were obtained and utilized as previously described [22]. Breast fibroblasts were cultured in M199 medium (Gibco, Grand Island, NY, USA) and Ham’s F12 (Gibco) supplemented with 10% heat-inactivated fetal bovine serum (FBS) (Gibco) and 1% Antibiotic-Antimycotic 100X (Gibco). HUVEC, MDA-MB-231 and MCF-7 cells (ATCC) were cultured in RPMI 1640 medium (Gibco) containing 5% FBS and 1% Antibiotic-Antimycotic. Cells were cultured at 37 °C in a humidified incubator with 5% CO_2_. Recombinant IL-6 was purchased from GenWay Biotech, San Diego, CA, USA.

### 2.2. RNA Purification and qRT-PCR

Total RNA was purified using the RNeasy Mini Kit (Qiagen, Manchester, UK) following the manufacturer’s recommendations and then was quantified using a Thermo Scientific NanoDrop ND-1000 Spectrophotometer. To prepare cDNA, RNA (1 μg) was amplified by RT-PCR using random primers (Clontech Laboratories-Advantage RT-for-PCR Kit) (Clontech Laboratories, Mountain View, CA, USA) following the manufacturers’ recommendations. Next, the Fast Start essential DNA Green Master 2X (Roche, New York, NY, USA) and specific primers were used for qRT-PCR, using the LightCycler 96 Real-Time PCR System (Roche). GAPDH expression levels were used for normalization, and gene expression differences were calculated using the threshold cycle (Ct). The sequences of primers are depicted in the Supplementary Table S1.

### 2.3. siRNA Transfection

DCN-siRNA and a scrambled sequence used as control were purchased from Santa Cruz, Santa Cruz, CA, USA. The transfections were performed utilizing the Lipofectamine RNAiMAX transfection reagent (Invitrogen, Carlsbad, CA, USA) using 30 nM siRNA incubated for 3 days.

### 2.4. Transfections with Plasmids

Plasmid transfections were performed utilizing a vector bearing the DCN-ORF and an empty vector (Origene, Rockville, MD, USA) (2 µg) using Lipofectamine 3000 Transfection Kit (Invitrogen) following the recommended protocol. Briefly, exponentially growing cells were transfected for 3 days. Puromycin (2 µg/mL) was utilized to select the transfected cells.

### 2.5. shRNA Transfection

Vectors bearing DCN-shRNA and control shRNA were purchased from (Santa Cruz). The transfections were performed utilizing the Lipofectamine 3000 Transfection Kit (Invitrogen) following the recommended protocol. Briefly, exponentially growing cells were transfected for 3 days. Puromycin (2 µg/mL) was utilized to select the transfected cells.

### 2.6. Cell Lysate Preparation and Immunoblotting

Whole cell lysates were prepared as previously described [23]. Briefly, cells were homogenized with RIPA buffer (Sigma-Aldrich, St. Louis, MI, USA) supplemented with protease inhibitors (Roche). The obtained cell lysates were centrifuged, and the resulting supernatants were stored at −80 °C. The extracted proteins were separated using SDS-PAGE and then were transferred to a polyvinylidene difluoride membrane (PVDF) (Bio-Rad, Hercules, CA, USA), which was first blocked with 5% powdered skimmed milk in TBST (Sigma-Aldrich) for 1 h and incubated with the appropriate primary antibody overnight and then an appropriate secondary antibody for 1 h. The visualization of the secondary antibody was performed using a chemiluminescence detection procedure according to the manufacturer’s protocol (Thermo Fisher Scientific). The used antibodies are depicted in Supplementary Tables S2 and S3.

### 2.7. Cell Invasion, Migration, and Proliferation Assays

Cell invasion, migration and proliferation capacities were assessed using the Real-Time Cell Analyzer–Dual Plat (RTCA-DP) xCELLigence System (ACEA Biosciences, Santa Clara, CA, USA) as per the manufacturer’s recommendations. In brief, cells (10^5^) in 100 μL of serum-free medium were loaded into the upper wells of CIM-Plate, while complete media (200 μL) were added to the lower chamber. For invasion, the microporous membranes were coated with matrigel (BD Biosciences, Franklin Lakes, NJ, USA) (20 μL). E-plate was used for the proliferation assay. The plates were incubated for 30 min at 37 °C in a 5% CO_2_ incubator and then were inserted into the RTCA-DP instrument inside the same incubator. The RTCA software (1.2.1) was utilized for data analysis.

### 2.8. Three-Dimensional Spheroid Assay

To generate spheroids, cells (2000 cells/well) were cultured in an ultralow-attachment 96-well plate using stem cell culture medium (171 medium supplemented with 1% ABM, 2% B-27, 20 ng/mL EGF, 500 ng/mL HC, 4% FBS and 5 μg/mL insulin) and then were incubated for several days until the formation of spheres. Only large spheres (diameter ≥ 100 µm) were counted with an inverted microscope (FLoid Cell Imaging Station, from OPTICA, Washington, DC, USA).

### 2.9. Serum-Free Conditioned Media Preparation

Cells were cultured in SFM (0.5% FBS) for 24 h, and then media were collected and centrifuged. The obtained SFCM were aliquoted and frozen at −80 °C until needed.

### 2.10. ELISA Assays

ELISA assays were carried out utilizing SFCM according to the manufacturer’s recommendations. Briefly, the plates were coated with the SFCM for 2 h and then were washed and incubated with the protein conjugate for 1 h. After washing, the substrate solution was added for 30 min, and then the stop solution was added and the plate was immediately read using a microplate (ELISA) reader (Bio-Rad) at 450 nm. VEGF Quantikine ELISA Kit, CXCL12/SDF-1 Quantikine ELISA Kit, CXCL8/IL-8 Quantikine ELISA Kit and IL-6 Quantikine ELISA Kit were obtained from R&D Systems, Minneapolis, MN, USA.

### 2.11. Human Cytokine Antibody Array

Human Cytokine Array C5 membranes (RayBiotech, Georgia, GA, USA) were first blocked with the blocking buffer and coated with the obtained SFCM overnight. Next, the membranes were washed and incubated with the biotinylated antibody cocktail and then with HRP-Streptavidin. The visualization of the HRP-Streptavidin was performed using the chemiluminescence detection buffers.

### 2.12. Orthotopic Tumor Xenografts

Experiments using animals were approved by the KFSH&RC Institutional Animal Care and Use Committee (ACUC) and were performed following relevant national and international guidelines. Orthotopic breast tumor xenografts were obtained by co-injecting MDA-MB-231 cells (2 × 10^6^) with NBF25-shRNA or NBF25-CTRL cells (4 × 10^6^) under the nipple of each mouse (*n* = 5). After tumor growth, a caliper was used to measure volumes, which were calculated according to the prolate ellipsoid formula: tumor volume = length × (width)^2^ × 0.5. Tumors were excised and were snap-frozen in liquid nitrogen.

### 2.13. Cytotoxicity Assay

Cells (5 × 10^3^) were first seeded in 96-well plates and then treated for 72 h. WST1 reagent (Roche) was added as described in the manufacturer’s protocol. The absorbance of the samples was recorded with a microplate (ELISA) reader (Bio-Rad) at 450 nm.

### 2.14. Statistical Analysis and Quantification

Student’s *t*-test was utilized for statistical analysis using GraphPad Prism software (4.7.8). *p*-values of 0.05 or less were considered as statistically significant. Quantification was performed using ImageJ (v 1.53). 

## 3. Results

### 3.1. Decorin Is Downregulated in Breast-Cancer-Associated Fibroblasts Relative to Their Adjacent Normal Counterparts

**Figure 1:**
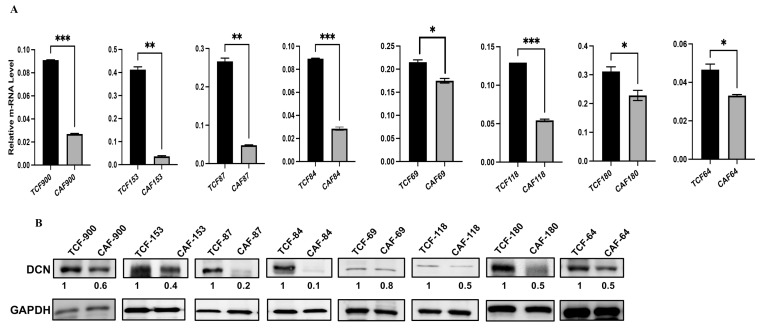
Decorin is downregulated in CAFs versus TCFs. (**A**) Total RNA was extracted from the indicated CAF/TCF pairs, and the level of the *DCN* mRNA was assessed by qRT-PCR. Error bars represent mean ± SD (*n* = 3). * *p* ≤ 0.05, ** *p* ≤ 0.01; *** *p* ≤ 0.001. (**B**) Whole cell lysates were prepared from the indicated cells and were used for immunoblotting analysis; GAPDH was used as internal control. The numbers below the bands represent expression fold change in CAFs relative to their corresponding TCFs after correction against the internal control GAPDH.

Decorin is secreted from normal fibroblasts, and this cytokine has non-cell-autonomous anti-cancer effects. Therefore, we decided to assess the level and test the role of DCN in breast stromal fibroblasts. To this end, the expression level of the gene was first assessed in eight human breast CAFs and their counterpart fibroblasts isolated from adjacent histologically normal breast cells (TCFs). CAF/TCF pairs were always used simultaneously at similar passages. Total RNA was purified from CAF/TCF pairs, and specific primers for *DCN* as well as *GAPDH* (used as internal control) were utilized for amplification by quantitative RT-PCR (qRT-PCR). Figure 1A shows a decrease in the *DCN* mRNA level in all CAFs, as compared to their adjacent TCFs (100%). To confirm this at the protein level in cancer-associated fibroblasts, whole cell extracts were prepared from the same TCF/CAF pairs, and specific anti-DCN and anti-GAPDH (used as internal control) antibodies were utilized for immunoblotting analysis. Figure 1B shows that the level of the DCN protein was lower in all CAFs (100%) as compared to their corresponding TCFs. This shows a positive correlation between the DCN mRNA and protein expression levels in all the TCF/CAF pairs, suggesting that the decrease in the DCN protein level in CAFs is taking place at the mRNA level.

### 3.2. Breast Cancer Cells and rIL-6 Downregulate DCN in Normal Breast Fibroblasts

**Figure 2:**
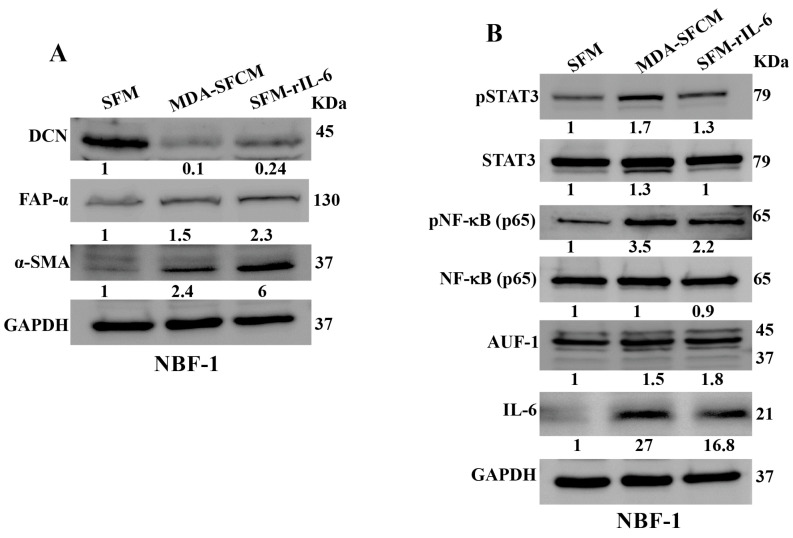
Breast cancer cells and rIL-6 downregulate DCN in normal breast fibroblasts. NBF-1 cells were cultured in SFM and used as a control (SFM), or in MDA-MB-231-SFCM (MDA-SFCM), or in SFM containing 3.5 ng/mL of the rIL-6 protein (SFM-rIL-6) for 24 h. (**A**,**B**) Whole cell lysates were prepared for immunoblotting analysis using specific antibodies against the indicated proteins. GAPDH was used as internal control. The numbers below the bands represent protein level fold change relative to the control (SFM) after correction against the internal loading control GAPDH.

Next, we wanted to know whether BC cells can downregulate DCN in breast fibroblasts and the role of IL-6 in this process. To this end, NBF-1 cells were incubated with either serum-free medium (SFM) or SFM containing rIL-6 (3.5 ng/mL) or serum-free conditioned medium (SFCM) collected from the highly invasive breast cancer cells MDA-MB-231 (SFM, SFM-rIL-6 and MDA-SFCM, respectively). Whole cell lysates were prepared and were used for immunoblotting analysis utilizing GAPDH as an internal control. The endogenous DCN level was reduced 10-fold and 4.1-fold in fibroblast cells treated with MDA-SFCM and SFM-rIL-6 compared to controls, respectively (Figure 2A). By contrast, the level of two main biomarkers of active fibroblasts α-SMA and FAP-α were increased in cells treated with MDA-SFCM and rIL-6 compared to controls (Figure 2A). This indicates that breast cancer cells and rIL-6 downregulated DCN and activated NBFs. To delineate the molecular pathway that mediates MDA-SFCM- and rIL-6-related DCN downregulation and activation of NBFs, we first tested the effect of these two factors on the two major transcription factors STAT3 and NF-κB. The levels of the active forms of both STAT3 and NF-κB increased in cells expressing low levels of DCN compared to controls, while the basal levels were not affected (Figure 2B). This led to the activation of the downstream effectors IL-6 and AUF-1, a master regulator of all activation biomarkers [8,24,25] (Figure 2B). This indicates that breast cancer cells can downregulate DCN and activate NBFs in an IL-6-dependent manner, through the activation of STAT3/NF-κB signaling.

### 3.3. Downregulation of DCN Activates Normal Breast Fibroblasts

**Figure 3:**
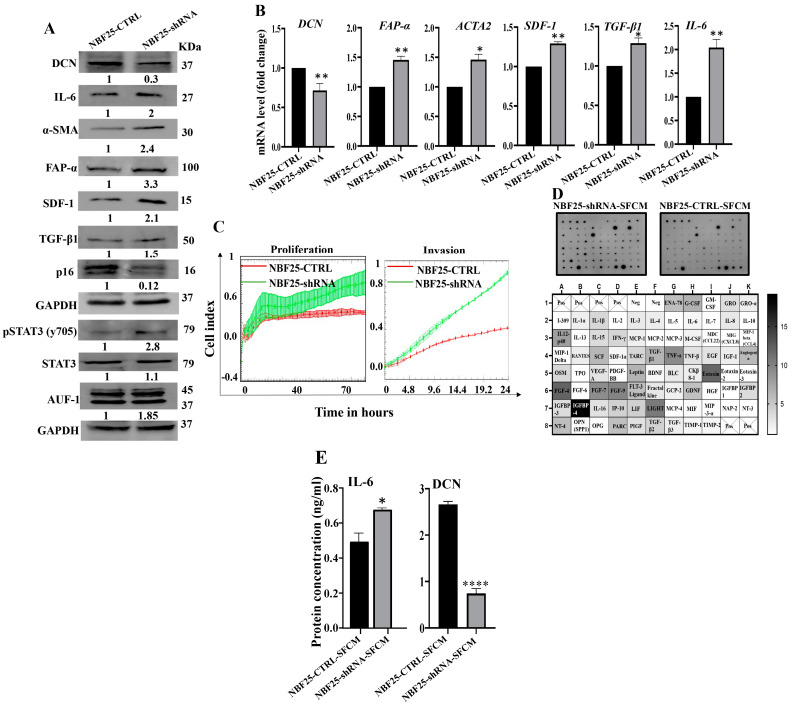
Downregulation of DCN activates normal breast fibroblasts. (**A**) NBF-25 cells were transfected with an empty vector (NBF25-CTRL) or a vector bearing the DCN-shRNA (NBF25-shRNA). Whole cell lysates were prepared and were used for immunoblotting analysis. The numbers below the bands represent fold change relative to the control (NBF25-CTRL) after correction against the internal control GAPDH. (**B**) Total RNA was prepared, and then the mRNAs of the indicated genes were amplified using qRT-PCR. Error bars represent mean ± SD (*n* = 3). * *p* ≤ 0.05, ** *p* ≤ 0.01. (**C**) Cell invasion and proliferation abilities were assessed using the RTCA-DP xCELLigence System. Data are representative of different experiments performed in triplicate. (**D**) SFCM from the NBF25-CTRL and NBF-25-shRNA cells were applied to the human cytokine antibody array membrane (C5). The intensities of the spots in the NBF25-shRNA-SFCM cytokine array membrane were quantified by densitometric analysis and divided by the values obtained in the control for each protein and were presented as fold change in the single gradient heatmap. (**E**) SFCM from the indicated cells were used to assess the level of the indicated proteins by ELISA (*n* = 3). Error bars represent mean ± SD. * *p* ≤ 0.05, **** *p* ≤ 0.0001.

Next, we sought to investigate the effect of DCN downregulation on normal breast fibroblasts. To this end, NBF-25 cells were transfected with either an empty vector or a vector bearing specific DCN-shRNA (NBF25-CTRL and NBF25-shRNA cells, respectively). NBF25-CTRL cells were used as a control. Figure 3A shows that the level of endogenous DCN protein was decreased 3-fold in NBF25-shRNA cells compared to the control. This was accompanied by a decrease in the level of the tumor suppressor p16 while the levels of the α-SMA, SDF-1, FAP-α, TGF-β1 and IL-6 proteins increased in NBF25-shRNA cells compared to the control (Figure 3A). Similar effects were observed at the mRNA levels of the genes *DCN*, *α-SMA*, *SDF-1*, *FAP-α*, *TGF-β1* and *IL-6* using qRT-PCR (Figure 3B). This shows that the downregulation of DCN in NBF-25 cells increased the level of several activation biomarkers at both the protein and mRNA levels.

Since AUF-1 controls the expression of all these active fibroblast biomarkers, we decided to test the effect of DCN downregulation on AUF-1. Figure 3A shows that AUF-1 was upregulated (1.85-fold) in DCN-deficient cells as compared to controls. This was mediated through the activation of the AUF-1 upstream activator STAT3 [8]. Indeed, the level of the phosphorylated form of STAT3 was increased in DCN-deficient cells compared to control cells, while the basal level of STAT3 was not affected (Figure 3A). This indicates that DCN downregulation activated NBFs through the STAT3/AUF-1 pathway.

Next, the effect of DCN downregulation on cell invasion and proliferation abilities of NBF25-CTRL and NBF25-shRNA cells was tested. To this end, cells were seeded in CIM-plates and E-plates and then were incubated for 24 h (invasion) or 72 h (proliferation) using the RTCA-DP xCELLigence System. Figure 3C shows that the invasion and proliferation capacities of NBF25-shRNA cells were higher compared to NBF25-CTRL cells, which confirmed that downregulation of DCN activates NBFs. To determine the effect of DCN downregulation on the secretion of various cancer-promoting cytokines, NBF25-CTRL and NBF25-shRNA cells were cultured in SFM for 24 h, and then SFCM were collected (NBF25-CTRL-SFCM and NBF25-shRNA, respectively) and were applied on the RayBiotech human cytokine array C-5. Figure 3D and Supplementary Figure S1 show that DCN downregulation increased the secreted levels of several pro-carcinogenic cytokines including IL-1α, IL-1β, TNF-α, SDF-1, TGF-β1 and IL-6. This was confirmed by the enzyme-linked immunosorbent assay (ELISA) for IL-6 and decorin (Figure 3E).

To confirm these results, DCN was downregulated using specific siRNA. Therefore, TCF-180 cells were transfected with either DCN siRNA or a scrambled sequence that was used as control. Total RNA was extracted, and the effects of DCN siRNA were assessed using qRT–PCR. Supplementary Figure S2A shows that DCN-siRNA caused a significant reduction in the expression level of the DCN mRNA. Subsequently, immunoblotting analysis showed that the DCN protein level was reduced in the DCNsiRNA-treated cells (TCF180-DCN-si) as compared to their corresponding control cells (TCF180-Ctrl-si) (Supplementary Figure S2B). Moreover, DCN downregulation increased the protein level of α-SMA, FAP-α, IL-6 and IL-8 (Supplementary Figure S2B). Interestingly, a similar effect was also obtained at the mRNA level for IL-6, ACTA2, CXCL12, TGF-β, FAP and IL-8 genes (Supplementary Figure S2B). DCN downregulation also enhanced the secretion of IL-6 (Supplementary Figure S2C). Next, the invasion and proliferation abilities of TCF180-DCN-si and TCF180-Ctrl-si cells were assessed using the RTCA-DP xCELLigence system, Supplementary Figure S2D shows that TCF180-DCN-si cells exhibited higher invasion and proliferation abilities than TCF180-Ctrl-si cells. These results confirm that DCN downregulation activates breast stromal fibroblasts.

### 3.4. DCN Downregulation in Normal Breast Fibroblasts Activates Their Paracrine Pro-EMT Effects

**Figure 4:**
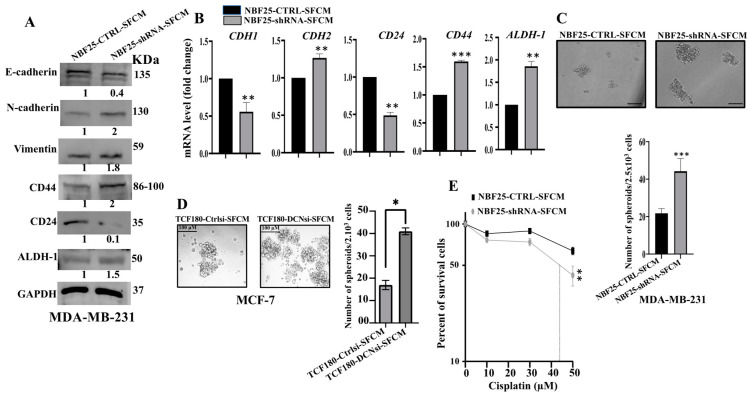
DCN downregulation in normal breast fibroblasts activates their paracrine pro-carcinogenic effects. Serum-free media (SFM) were added to either NBF25-CTRL or NBF25-shRNA cells and incubated for 24 h, and then SFCM were collected (NBF25-CTRL-SFCM and NBF25-shRNA-SFCM) and were applied on MDA-MB-231 cells for 24 h. (**A**) Whole cell lysates were prepared and were used for immunoblotting analysis. The numbers below the bands represent fold change relative to the control (NBF25-CTRL-SFCM) after correction against the internal control GAPDH. (**B**) Total RNA was prepared, and then the mRNA levels of the indicated genes were assessed using qRT-PCR. Error bars represent mean ± SD (*n* = 3). ** *p* ≤ 0.01, *** *p* ≤ 0.001. (**C**,**D**) Cells (2 × 10^3^) were seeded in an ultralow-attachment 96-well plate containing the stem-cell-specific culture medium. The formed tumor spheres (≥100 μm) were counted. Representative photographs of tumor spheres. Scale bar = 100 μm. Histograms depicting the number of the formed tumor spheres. Error bars represent mean ± SD; *n* = 3. * *p* ≤ 0.05, *** *p* ≤ 0.001. (**E**) MDA-MB-231 cells were seeded in 96-well plates (5.10^3^), and then SFCM from NBF25-CTRL or NBF25-shRNA cells were added and cells were incubated for 24 h. Then, different concentrations of cisplatin were added for 72 h. Cell viability was measured using the WST-1 assay. Error bars represent mean ± SD; *n* = 3. ** *p* ≤ 0.01.

Next, we decided to investigate the effect of DCN downregulation on the paracrine pro-carcinogenic effects of fibroblasts on breast cancer cells. Therefore, SFCM were collected from NBF25-CTRL and NBF25-shRNA cells (NBF25-CTRL-SFCM and NBF25-shRNA-SFCM, respectively) and were used to treat MDA-MB-231 breast cancer cells for 24 h. Then, whole cell lysates were prepared and used for immunoblotting analysis. Figure 4A shows that SFCM from NBF25-shRNA cells activated the epithelial-to-mesenchymal transition (EMT) process in MDA-MB-231-breast cancer cells, via downregulation of the epithelial marker E-cadherin and upregulation of the mesenchymal markers N-cadherin and vimentin as compared to controls. This effect on the EMT markers was confirmed at the mRNA level of the E-cadherin- and N-cadherin-coding genes (*CDH1* and *CDH2*, respectively). Indeed, while the epithelial marker *CDH1* mRNA level was reduced 2-fold, the level of the mesenchymal marker *CDH2* was increased 1.5-fold in the MDA-MB-231 cells treated with NBF25-shRNA-SFCM relative to controls (Figure 4B).

Similarly, SFCM from TCF180-DCNsi cells (TCF180-DCNsi-SFCM) increased the level of the mesenchymal markers N-cadherin, Twist and vimentin relative to their levels in control cells (Supplementary Figure S3A). On the other hand, the levels of the epithelial markers E-cadherin and EpCAM were decreased, as compared to control cells (Supplementary Figure S3A). Moreover, the invasion and proliferation capacities of MCF-7 cells were higher in the presence of TCF180-DCNsi-SFCM than in the presence of TCF180-Ctrlsi-SFCM (Supplementary Figure S3B). This indicates that DCN-deficient breast fibroblasts can promote the EMT process in BC cells in a paracrine manner.

### 3.5. DCN Downregulation in Normal Breast Fibroblasts Activates Their Paracrine Pro-Stemness Effects

Next, we investigated the effect of NBF-25-shRNA-SFCM on the stemness features of MDA-MB-231 breast cancer cells. Figure 4A shows a clear upregulation of CD44 (2-fold) and ALDH-1 (1.5-fold), while CD24 was downregulated (10-fold) in MDA-MB-231 cells treated with NBF25-shRNA-SFCM relative to control cells. This was confirmed at the mRNA level (Figure 4B). To further investigate the paracrine effect of DCN downregulation in NBF cells on the stemness features of MDA-MB-231 cancer cells, we treated MDA-MB-231 cells with SFCM from NBF25-CTRL or NBF25-shRNA cells, and then treated cells were seeded in an ultralow-attachment 96-well plate containing a stem-cell-specific medium. Ten days later, spheroids with size ≥ 100 μm were counted. The MDA-MB-231 cells treated with NBF-25-shRNA-SFCM exhibited a higher number of spheroids relative to the control cells (Figure 4C). Likewise, TCF180-DCNsi-SFCM upregulated CD44 in MCF-7 cells as compared to controls (Supplementary Figure S3A). Also, TCF180-DCNsi-SFCM promoted the formation of spheroids in MCF-7 cells more than TCF180-Ctrlsi-SFCM (Figure 4C). These results indicate that DCN-deficient breast stromal fibroblasts promote carcinogenesis through paracrine induction of EMT and stemness in BC cells.

### 3.6. DCN-Deficient Fibroblasts Promote Chemoresistance in Breast Cancer Cells

Next, since cancer stem cells (CSCs) play a critical role in cancer recurrence and resistance to drugs, we sought to assess the effect of DCN-deficient fibroblasts on the response of BC cells to chemotherapeutic drugs. To this end, we incubated MDA-MB-231 breast cancer cells (5.10^3^) with SFCM from NBF25-shRNA or NBF25-CTRL for 24 h. Then, cells were treated with different concentrations of cisplatin (0, 10, 30 and 50 µM) for 72 h, and the WST1 assay was performed to evaluate the response of cells to the drug. Figure 4E shows that the treatment of MDA-MB-231 cells with SFCM from NBF25-shRNA cells enhanced their survival compared to controls. This indicates that DCN-deficient fibroblasts promote the resistance of BC cells to cisplatin in a paracrine fashion.

### 3.7. DCN-Deficient Breast Fibroblasts Induce Tumor Growth and Stemness In Vivo

**Figure 5:**
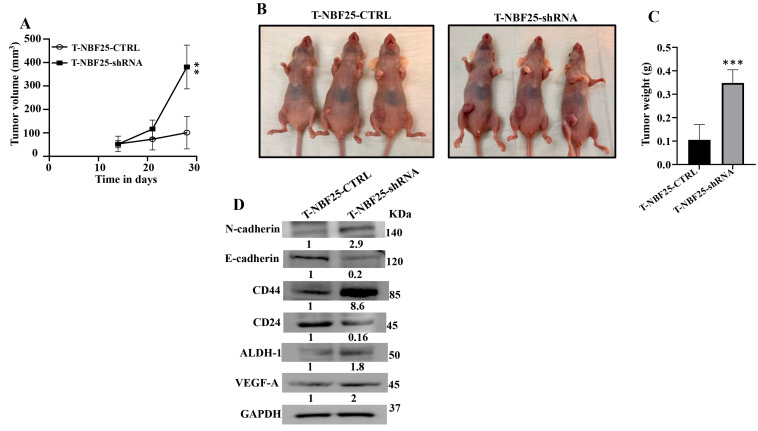
DCN-deficient breast fibroblasts induce tumor growth in vivo. Breast cancer orthotopic tumor xenografts were created by injecting MDA-MB-231 cells in combination with either NBF25-CTRL or with NBF25-shRNA cells (T-NBF25-CTRL and T-NBF25-shRNA, respectively) under the nipple of female nude mice. (**A**) Tumor volumes; error bars represent ** *p* ≤ 0.01. (**B**) Pictures of animals bearing tumors within the nipples. (**C**) Tumors were excised and weighed (g); *** *p* ≤ 0.001. (**D**) Whole cell lysates were prepared from excised tumors and were used for immunoblotting analysis. The numbers below the bands represent fold change relative to the control (T-NBF25-CTRL) after correction against the internal control GAPDH.

To confirm the paracrine pro-carcinogenic effects of DCN-deficient fibroblasts, we sought to test the effect of these cells on BC tumor growth in vivo. Therefore, MDA-MB-231 cells (2.10^6^) in combination either with NBF25-CTRL or with NBF25-shRNA cells (4.10^6^) (*n* = 5 for each inoculation) were injected under the nipple of nude mice. Both inoculations generated tumors in only three out of five animals. Figure 5A shows that the presence of NBF25-shRNA cells promoted tumor growth compared to controls in a time-dependent manner. Figure 5B shows that all tumors bearing NBF25-shRNA cells were much bigger than those bearing control cells. Tumors were excised and were weighed. Tumors containing DCN-deficient fibroblasts had higher tumor weights compared to controls (Figure 5C). Next, whole cell lysates were prepared from tumors containing NBF25-CTRL and tumors bearing NBF25-shRNA. Figure 5D shows that like what has been shown in vitro, the presence of NBF-shRNA cells promoted EMT and stemness in breast cancer cells. Indeed, the presence of NBF25-shRNA upregulated the mesenchymal marker N-cadherin and downregulated the epithelial marker E-cadherin. Furthermore, CD44 and ALDH1 were upregulated while CD24 was downregulated. This indicates that DCN-deficient fibroblasts promote tumor growth as well as the pro-metastatic processes EMT and stemness in vivo as well.

### 3.8. DCN-Deficient Fibroblasts Promote Angiogenesis In Vitro and In Vivo

**Figure 6:**
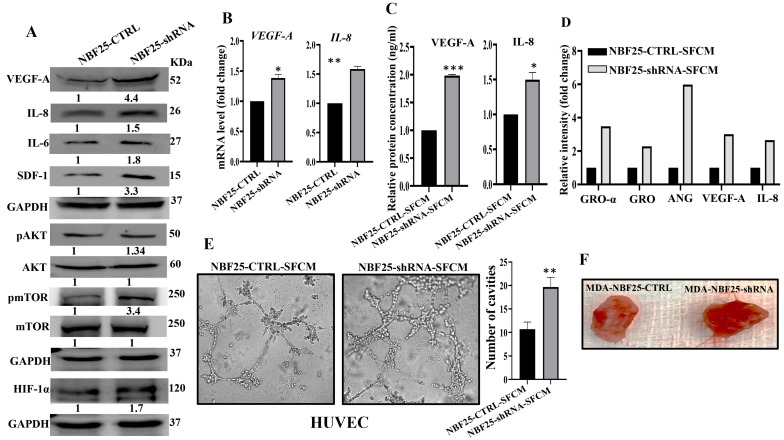
Downregulation of DCN enhances the pro-angiogenic effects of breast fibroblasts. (**A**) NBF-25 cells were transfected with an empty vector (NBF25-CTRL) or a vector bearing the DCN-shRNA (NBF25-shRNA). Whole cell lysates were prepared and were used for immunoblotting analysis. The numbers below the bands represent fold change relative to the control (NBF25-CTRL) after correction against the internal control GAPDH. The levels of phosphorylated proteins were normalized against the total amount of their relative non-phosphorylated forms (**B**), Total RNA was prepared, and then the mRNAs of the indicated genes were amplified using qRT-PCR. Error bars represent mean ± SD (*n* = 3). * *p* ≤ 0.05, ** *p* ≤ 0.01. (**C**) SFCM from the indicated cells were used to assess the level of the indicated proteins by ELISA (*n* = 3). Error bars represent mean ± SD. * *p* ≤ 0.05, *** *p* ≤ 0.001. (**D**) SFCM from NBF25-CTRL and NBF-25-shRNA cells were applied to the human cytokine antibody array membrane (C5). The histogram shows fold changes relative to the respective controls for each indicated protein. (**E**) A 96-well plate was coated with matrigel and incubated for 1 h, and then HUVEC cells were seeded in the presence of NBF25-CTRL-SFCM or NBF25-shRNA-SFCM and then were incubated for 4 h. Representative photographs of HUVEC cavities are shown (left panel). The number of cavities was calculated and presented as a histogram. Error bars represent mean ± SD (*n* = 3), ** *p* ≤ 0.01. (**F**). Photographs of pieces of excised and cut tumors.

Active fibroblasts promote the formation of new blood vessels, which help tumors to spread and grow [6]. Therefore, we sought to investigate the role of DCN downregulation in inducing the pro-angiogenic capacity of breast fibroblasts (BFs). To do this, we first assessed the effect of DCN downregulation on the expression of the most important angiogenic factors. Figure 6A shows that DCN downregulation in NBFs increased the expression of VEGF-A, IL-8, IL-6 and SDF-1. These results were confirmed at the mRNA level of the two major angiogenic factor genes *VEGF-A* and *IL-8* (Figure 6B). Furthermore, the secreted level of both proteins VEGF-A and IL-8 was also higher in DCN-deficient fibroblasts compared to control cells (Figure 6C). Also, the analysis of the cytokine array (depicted in Figure 3D) showed that DCN-deficient cells secreted higher levels of several pro-angiogenic cytokines such as GRO-α, GRO and Angiogenin (Figure 6D). These findings prompted us to analyze the effect of DCN downregulation on angiogenesis in vitro by assessing the differentiation of endothelial cells into cavities. To this end, SFCM collected either from NBF25-CTRL or NBF25-shRNA cells were used to treat HUVEC cells (40 × 10^3^) that were seeded on a matrigel-coated 96-well plate and then were incubated for 4 h. The formed cavities were counted. Figure 6E shows that the number of HUVEC cells that were differentiated into close cavities was significantly higher in the presence of SFCM from NBF25-shRNA cells compared to the controls. Figure 6F shows that tumor xenografts that contain DCN-deficient fibroblasts exhibited higher blood vessel density compared to the corresponding controls. These results prompted us to ask how DCN-deficient breast fibroblasts promote the differentiation of endothelial cells and the formation of blood vessels. To this end, we tested the effect of DCN downregulation on the major angiogenesis signaling pathway AKT/mTOR/HIF-1α, through enhancing the secretion of VEGF-A [26]. Figure 6A shows that DCN downregulation activated AKT and mTOR. In fact, while the basal levels of AKT and mTOR were not affected, DCN downregulation increased the level of the active/phosphorylated forms of both proteins compared to controls (Figure 6A). Additionally, HIF-1α, a downstream trans-activator of mTOR, increased in DCN-deficient cells compared to controls (Figure 6A). This indicates that the downregulation of DCN in breast fibroblasts induces angiogenesis through activation of AKT/mTOR/HIF-1α and the consequent increase in the secretion of several pro-angiogenic factors such as VEGF-A and IL-8.

### 3.9. DCN Inhibits the Active Features of Breast-Cancer-Associated Fibroblasts

**Figure 7:**
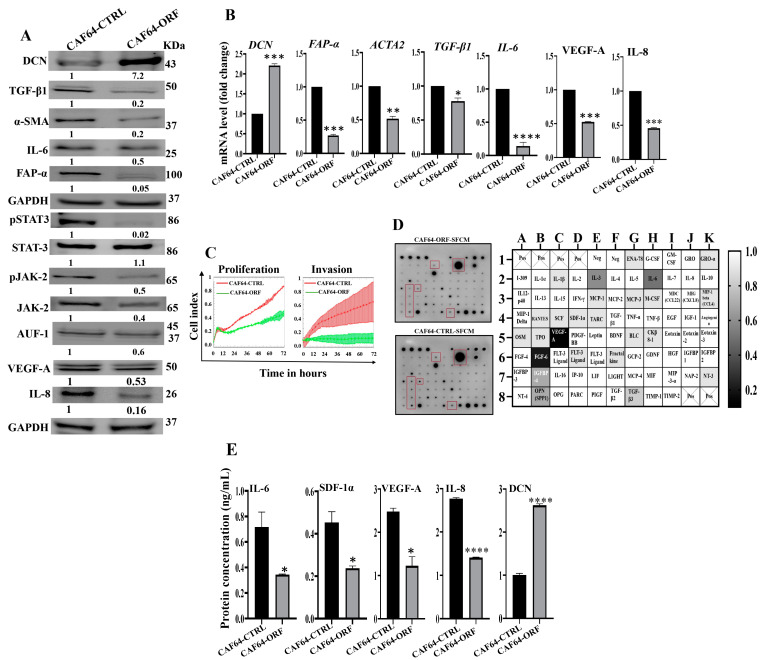
DCN upregulation inhibits the active features of breast-cancer-associated fibroblasts. (**A**) CAF-64 cells were transfected with an empty vector (CAF64-CTRL) or a vector bearing the DCN-ORF (CAF64-ORF). Whole cell lysates were prepared and were used for immunoblotting analysis. The numbers below the bands represent fold change relative to the control (CAF64-CTRL) after correction against the internal control GAPDH. The levels of phosphorylated proteins were normalized against the total amount of their relative non-phosphorylated forms. (**B**) Total RNA was prepared, and then the mRNAs of the indicated genes were amplified using qRT-PCR. Error bars represent mean ± SD (*n* = 3). * *p* ≤ 0.05, ** *p* ≤ 0.01, *** *p* ≤ 0.001, **** *p* ≤ 0.0001. (**C**) Cell invasion and proliferation abilities were assessed using the RTCA-DP xCELLigence System. Data are representative of different experiments performed in triplicate. (**D**) SFCM from the indicated cells were applied onto the human cytokine antibody array membrane (C5). The highly differentially expressed cytokines are highlighted. The histogram shows fold changes relative to the respective controls for each indicated protein. (**E**) SFCM from the indicated cells was used to assess the level of the indicated proteins by ELISA (*n* = 3). Error bars represent mean ± SD. * *p* ≤ 0.05, **** *p* ≤ 0.0001.

After showing DCN downregulation-dependent activation of breast stromal fibroblasts, we decided to investigate the effect of DCN upregulation on active breast CAFs. Therefore, we first transfected CAF-64 cells with either an empty vector or a vector bearing the DCN open reading frame (ORF), which generated CAF64-CTRL and CAF64-ORF cells, respectively. CAF64-CTRL cells were used as a control. Whole cell lysates were prepared and used for immunoblotting analysis utilizing GAPDH as an internal control. As expected, the level of the DCN protein was increased (7-fold) in the CAF64-ORF cells relative to the CAF64-CTRL cells (Figure 7A). On the other hand, the levels of the biomarkers of active CAF cells (α-SMA, FAP-α, IL-6, TGF-β1) were decreased in the CAF64-ORF cells compared to controls (Figure 7A). Likewise, the *DCN* mRNA level was higher in CAF64-ORF cells relative to CAF64-CTRL cells (Figure 7B). However, the mRNA level of the other genes (*α-SMA*, *TGF-β1*, *FAP-α* and *IL-6*) was decreased in CAF64-ORF cells compared to CAF64-CTRL cells (Figure 7B). This indicates DCN-dependent suppression of active CAF biomarkers. To confirm this, we tested the effect of DCN upregulation on cell invasion and proliferation abilities of CAF-64 cells. To this end, cells were seeded in CIM-plates and E-plates and then were incubated for 72 h. An analysis of the RTCA-DP xCELLigence System generated results shows that the invasion and proliferation capacities of CAF64-ORF cells were inhibited compared to CAF64-CTRL cells (Figure 7C). This shows that DCN upregulation inhibits the proliferative/invasive capacities of active CAF cells. To determine the effect of DCN upregulation on the secretion of cancer-promoting cytokines, CAF64-ORF and CAF64-CTRL cells were cultured in SFM for 24 h, and then SFCM were collected and were applied on the RayBiotech human cytokine array C-5. Figure 7D shows that the upregulation of DCN in CAF-64 cells decreased the secreted level of several pro-carcinogenic cytokines, including SDF-1, TGF-β1 and IL-6, and also the pro-angiogenic factor VEGF-A. This was confirmed by ELISA for the pro-angiogenic and metastatic factors SDF-1, IL-6, IL-8 and VEGF-A (Figure 7E). This figure also confirms the increase in the secretion of DCN in CAF64-ORF cells relative to controls. The effect on VEGF-A and IL-8 was also confirmed at the mRNA and protein levels, confirming DCN-dependent negative control of VEGF-A (Figure 7A and Figure 7B, respectively). This confirms the inhibitory effect of DCN upregulation on the active features of breast-cancer-associated fibroblasts (Figure 7E).

These findings raised an important question on how DCN modulates the expression of all these active CAF biomarkers, leading to inhibition of the active features of CAFs. To address this, we investigated the effect of DCN upregulation on the expression of AUF-1, a master regulator of all these CAF biomarkers [8,24,25]. Figure 7A shows that DCN upregulation reduced the expression of AUF-1. To delineate the molecular mechanism underlying this action, we tested the effect of DCN on STAT3, an upstream trans-activator of AUF-1. Indeed, DCN upregulation inactivated STAT3 as well as its upstream protein kinase JAK-2 (Figure 7A). This indicates that DCN suppresses the active features of CAFs by suppressing the STAT3/AUF-1 pathway, a major activator of breast stromal fibroblasts.

### 3.10. DCN Upregulation Inhibits the Paracrine Pro-EMT and Pro-Stemness Effects of Active CAFs

**Figure 8:**
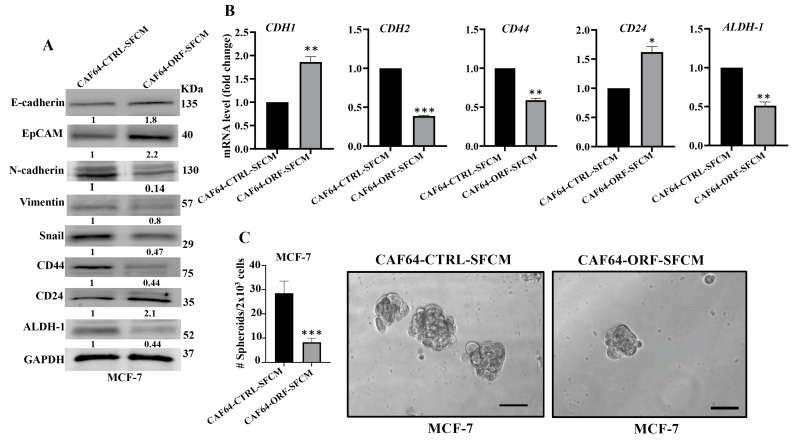
DCN upregulation inhibits the paracrine pro-carcinogenic effects of active CAFs. SFM was added to either CAF64-CTRL or CAF64-ORF cells and incubated for 24 h, and then SFCM were collected (CAF64-CTRL-SFCM and CAF64-ORF-SFCM, respectively) and then were applied on MCF-7 cells for 24 h. (**A**) Whole cell lysates were prepared and were used for immunoblotting analysis. The numbers below the bands represent fold change relative to the control (CAF64-CTRL-SFCM) after correction against the internal control GAPDH. (**B**) Total RNA was prepared, and then the mRNA levels of the indicated genes were assessed using qRT-PCR. Error bars represent mean ± SD (*n* = 3). * *p* ≤ 0.05, ** *p* ≤ 0.01, *** *p* ≤ 0.001. (**C**) Cells (2 × 10^3^) were seeded in an ultralow-attachment 96-well plate containing the stem-cell-specific culture medium. The formed tumor spheres (≥100 μm) were counted. Representative photographs of tumor spheres (right panel). Scale bar = 100 μm. Histograms depict the number of the formed tumor spheres (left panel). Error bars represent mean ± SD (*n* = 3). *** *p* ≤ 0.001.

After showing DCN-dependent inhibition of the secretion of several pro-carcinogenic cytokines from active CAF cells, we decided to investigate the effect of DCN upregulation on the paracrine pro-carcinogenic effects of CAFs on breast cancer cells. Therefore, SFCM were collected from CAF64-CTRL and CAF64-ORF cells (CAF64-CTRL-SFCM and CAF64-ORF-SFCM, respectively) and were used to treat MCF-7 breast cancer cells for 24 h. Then, whole cell lysates were prepared and were used for immunoblotting analysis utilizing GAPDH as an internal control. Figure 8A shows that CAF64-ORF-SFCM inhibited the epithelial-to-mesenchymal transition (EMT) process in MCF-7-breast cancer cells via upregulation of the epithelial markers (E-cadherin and EpCAM) and downregulation of the mesenchymal markers (vimentin and Snail) as compared to controls. This effect on the EMT markers was confirmed at the mRNA level of the *CDH1* and *CDH2* genes. Indeed, the *CDH1* mRNA level was increased 1.8-fold, while the mRNA level of the mesenchymal marker *CDH2* was reduced 2.5-fold in the MCF-7 cells treated with SFCM from CAF64-ORF cells relative to the controls (Figure 8B).

Next, we investigated the effect of CAF64-ORF-SFCM on the stemness features of MCF-7 breast cancer cells. Figure 8A shows a clear downregulation of CD44 and ALDH-1, while CD24 was upregulated in MCF-7 cells treated with CAF64-ORF-SFCM relative to CAF64-CTRL-SFCM. This was confirmed at the mRNA level (Figure 8B). To further investigate the paracrine effect of DCN upregulation in CAF cells on the stemness features of MCF-7 cancer cells, we treated MCF-7 cells with SFCM from CAF64-CTRL or CAF64-ORF cells, and then the treated cells were seeded in an ultralow-attachment 96-well plate containing a stem-cell-specific medium. Ten days later, spheroids with size ≥ 100 μm were counted. The MCF-7 cells treated with CAF64-ORF-SFCM exhibited a clear decrease in the number of spheroids relative to the control cells (CAF64-CTRL-SFCM). These results indicate that upregulation of DCN in CAF cells suppresses their pro-EMT and pro-stemness capacities. Together, these findings show that DCN upregulation suppresses the active features of breast CAFs and their pro-carcinogenic effects.

## 4. Discussion

Decorin, a potent multifunctional oncosuppressive proteoglycan, is mainly synthesized and deposited by fibroblasts [27,28]. In the present study, we have first shown that most CAFs express decorin at a lower level than their adjacent TCFs, at both the mRNA and protein levels. Likewise, a significant decrease in the level of DCN was previously observed within the stroma of various solid tumors including breast tumors [27,29]. This downregulation of DCN in CAFs suggested the possible role of cancer cells in downregulating DCN in the adjacent fibroblasts. Indeed, we have shown that the triple-negative breast cancer cells MDA-MB-231 reduced the level of DCN and increased the level of α-SMA and FAP-α in normal breast fibroblasts in a paracrine manner. This indicates that breast cancer cells can downregulate DCN in stromal fibroblasts through paracrine signaling. To delineate the signaling molecule, we have shown that, like SFCM from MAD-MB-231 cells, rIL-6 also reduced the level of DCN in NBFs. This suggests that IL-6 may play a key role in BC-dependent downregulation of DCN in BSFs. This was accompanied by an increase in the level of the major active fibroblasts’ biomarkers: FAP-α, α-SMA and IL-6, as well as their upstream activators: STAT3, NF-κB and AUF-1. This may indicate that BC cells could activate stromal fibroblasts through the secretion of IL-6 and the consequent activation of the IL-6/STAT3/AUF-1 pathway and the downregulation of DCN via a possible feed-forward mechanism. This shows the importance of DCN downregulation in stromal cells during breast carcinogenesis [28,30]. However, Hosoya et al. have recently shown that while stromal DCN expression was reduced in breast cancer with stage I, it was elevated paradoxically in that with higher stages (II and III) [31]. Nonetheless, this study has several limitations such as the cross-sectional observational nature of the study and the limited number of patients from a single center.

Therefore, it was important to elucidate the role of DCN downregulation in the activation of NBFs. To this end, we first downregulated DCN using specific siRNA and shRNA and showed that this specific reduction in the level of DCN activates NBFs. Indeed, DCN downregulation decreased the level of the tumor suppressor p16 and upregulated the major biomarkers of active fibroblasts FAP-α, α-SMA, SDF-1, TGF-β1 and IL-6 at both the mRNA and protein levels. Similarly, it has been shown that DCN controls the expression of α-SMA in human lung myofibroblast cell lines [32] and negatively regulates TGF-β1 in Chinese hamster ovary cells and human mesangial cells [33,34]. On the other hand, the upregulation of DCN in CAFs normalized the active traits of these cells and inhibited their proliferative and invasive capacities. Similarly, transfection of the invasive breast cancer cells (MDA-MB-231) with DCN resulted in the suppression of the carcinogenesis features of these cells [35]. Several other studies have also shown the role of DCN in suppressing cellular proliferation in other types of cells such as Chinese hamster ovary cells and arterial smooth muscle cells [34,36]. Furthermore, it has been shown that ectopic expression of DCN in a variety of cells such as glioma cells, colon cancer cells and inflammatory breast cancer cells inhibited the growth and the invasive capacities of these tumor cells [15,37,38].

**Figure 9:**
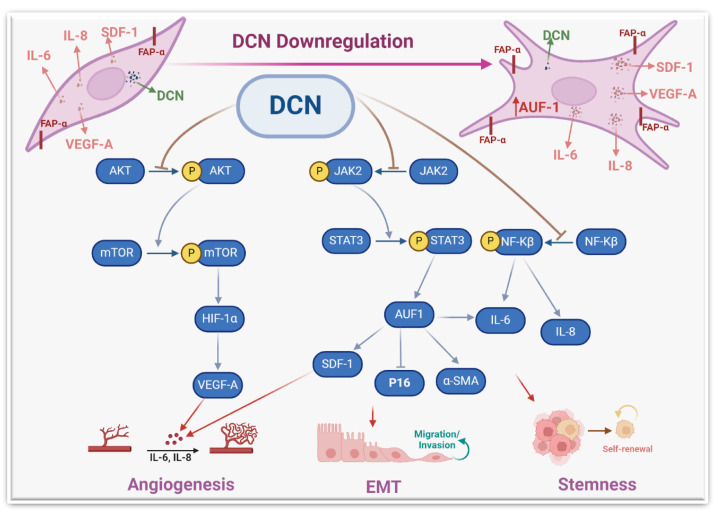
Schematic representation of the role of DCN in repressing the active features of breast stromal fibroblasts. More details are present in the text (Section 4).

These results indicate that DCN controls the expression of these fibroblast active biomarkers in various types of cells. Therefore, we wanted to understand how this cytokine can regulate the expression of these genes. We have shown that DCN suppresses STAT3 and its downstream effector AUF-1, which controls the expression of all these genes: IL-6, α-SMA, TGF-β1 and p16 [8]. Therefore, DCN could regulate the expression of these genes through the IL-6/STAT3/AUF-1 pathway (Figure 9).

The activation of these genes/pathways was accompanied by an increase in the proliferative and invasive capacities of stromal fibroblasts. Likewise, DCN knockout in murine embryonic fibroblasts enhanced their proliferative rate relative to wild-type cells [20]. Also, DCN knockout significantly induced cell invasion in glioma cell lines [39]. Together, these findings indicate that DCN has the capacity to repress the proliferative and invasive capacities of various types of cells, including breast stromal fibroblasts. This could be mediated through the inhibition of the STAT3 and NF-κB pro-carcinogenic pathways.

To further confirm DCN-downregulation-dependent activation of NBFs, we have shown that DCN downregulation induces the pro-carcinogenic features of NBFs in a paracrine manner. Indeed, SFCM from DCN-deficient fibroblasts promoted the EMT process, the proliferative/invasive capacities, and the mammary stemness-related features in BC cells in vitro. We have also shown that DCN downregulation promotes tumor growth in orthotopic tumor xenografts. This indicates that DCN downregulation in stromal fibroblasts can activate breast carcinogenesis and enhance the pro-invasive and metastatic potential of BC cells by promoting EMT and stemness (Figure 9). This could be mediated through a concomitant decrease in the expression of the anti-carcinogenic cytokine (DCN) and an increase in several pro-carcinogenic cytokines such as IL-6, SDF-1 and TGF-β. These findings explain the decrease in the level of DCN in breast stromal cells. Indeed, it has been found that DCN level was expressed in the stroma of normal tissues while it was significantly reduced or absent in the corresponding tumors, which facilitated tumor growth and progression [29]. In another study, Zhang et al. have shown a gradual DCN downregulation in CAFs during the progression of hepatocellular carcinoma [40].

The paracrine pro-stemness effect of DCN-deficient fibroblasts was confirmed by showing that SFCM from DCN-deficient fibroblasts increased the resistance of BC cells to cisplatin. In fact, a meta-analysis study has shown that the low expression of stromal DCN predicts a bad prognosis in breast cancer patients [41]. This suggests that DCN downregulation in breast stromal fibroblasts can promote carcinogenesis and the resistance of cancer cells to therapy.

The other important pro-carcinogenic/metastatic process is angiogenesis, which is promoted and enhanced by active CAFs [6]. We have shown that DCN downregulation in BSFs promotes the differentiation of endothelial cells into cavities in a paracrine manner. This was confirmed by showing that DCN downregulation increases the expression and secretion of the main pro-angiogenic molecules VEGF-A, IL-8, IL-6 and SDF-1. On the other hand, DCN ectopic expression repressed the expression and secretion of SDF-1, IL-6, IL-8 and VEGF-A. It has been previously shown that CAFs promote vascular invasion of hepatocellular carcinoma through DCN downregulation [40]. To further confirm the role of DCN in angiogenesis and delineate the signaling pathway, we have shown that DCN downregulation activates the AKT signaling and its downstream protein kinase mTOR and its major effector HIF-1α (Figure 6). This indicates that DCN downregulation activates the pro-angiogenic potential of BSFs through the activation of the AKT signaling pathway and its downstream pro-angiogenic effectors VEGF-A, IL-8, IL-6 and SDF-1 (Figure 9). Similarly, it has been previously shown that decorin negatively regulates the expression of VEGF-A and HIF-1α in breast cancer cells [42].

## 5. Conclusions

The present findings show that DCN suppresses the active features of breast stromal fibroblasts and their paracrine pro-carcinogenic effects both in vitro and in animal models. Thereby, DCN could be of great therapeutic value for BC patients through targeting active CAFs, cancer cells and angiogenesis.

## Data Availability

The datasets used and/or analyzed during the current study are available from the corresponding author on reasonable request.

## Supplementary Materials

The following supporting information can be downloaded at: https://www.mdpi.com/article/10.3390/cells13080680/s1, Figure S1: Decorin downregulation in breast stromal fibroblasts enhances the secretion of several cytokines; Figure S2: Decorin downregulation activates breast stromal fibroblasts; Figure S3: Decorin down-regulation enhances the procarcinogenic effects of tumor counterpart fibroblasts; Table S1: List of primers; Table S2: List of primary antibodies; Table S3: List of secondary antibodies.
