# Supplementary material for: Decorin (DCN) Downregulation Activates Breast Stromal Fibroblasts and Promotes Their Pro-Carcinogenic Effects through the IL-6/STAT3/AUF1 Signaling
Source: Cells. 2024 Apr 14;13(8):680. doi: 10.3390/cells13080680 (PMC11049637; doi:10.3390/cells13080680)
Supplement: Supplementary file 1 [file cells-13-00680-s001.zip › cells-2871997-supplementary.pdf]

Supplementary Table S1. List of primers

Primers	Sequence	
GAPDH	*Forward*	*5'-GAGTCCACTGGCGTCTTC-3'*
	Reverse	*5'-GGGGTGCTAAGCAGTTGGT-3'*
DCN	*Forward*	*5'-TCA AAA GGT CAA GAT CAG CCC-3'*
	Reverse	*5'-CAC TAG CTT TGT GGG CAG TT-3'*
ACTA2 (α-SMA)	*Forward*	*5'-CTATGCCTCTGGACGCACAACT -3'*
	Reverse	*5'-CAGATCCAGACGCATGATGGCA -3'*
CXCL12 (SDF-1)	*Forward*	*5'- CTCAACACTCCAAACTGTGCCC -3'*
	Reverse	*5'-CTCCAGGTACTCCTGAATCCAC-3'*
TGF-β1	*Forward*	*5'-TACCTGAACCCGTGTTGCTCTC -3'*
	Reverse	*5'-GTTGCTGAGGTATCGCCAGGAA -3'*
IL-6	*Forward*	*5'-AGACAG CCA CTC ACC TCT TCA G -3'*
	Reverse	*5'- TTC TGC CAG TGC CTC TTT GCT G -3'*
IL-8	*Forward*	*5'- GAT CCA CAA GTC CTT GTT CCA -3'*
	Reverse	*5'- GCT TCC ACA TGT CCT CAC AA -3'*
AUF-1	*Forward*	*5'-GATCAAGGGGTTTTGGCTTT -3'*
	Reverse	*5'-GTTGTCCATGGGGACCTCTA-3'*
FAP-α	*Forward*	*5'-TGA CCA GAA CCA CGG CTT AT -3'*
	Reverse	*5'- AGC AAA CTG TCT GAG GGG TT*
CDH1	*Forward*	*5'-CCC GCC TTA TGA TTC TCT GCT CGT G-3'*
	Reverse	*5'-TCC GTA CAT GTC AGC CAG CTT CTT G-**3'***
CDH2	*Forward*	*5'-CCT CCA GAG TTT ACT GCC ATG AC-3'*
	Reverse	*5'-GTA GGA TCT CCG CCA CTG ATT C-3'*
ALDH1A1	*Forward*	*5'-TCT CTA TTT CTC TCC CCT CCC T-3'*
	Reverse	5'-ACC ATC TTT GAA GGG TTG GC-3'
VEGF-A	*Forward*	5′-CCCACTGAGGAGTCCAACAT-3′
	Reverse	5′-TGGATGGTGGTACAGTCAGAGC-3′
CD24	*Forward*	*5'- GAGAGATAACCCTGCCCGAG-3'*
	Reverse	*5'- AAAAGAAAAGTCCGCGCCTC -3'*
CD44	*Forward*	*5'-CCA GAA GGA ACA GTG GTT TGG C-3'*
	Reverse	*5'-ACT GTC CTC TGG GCT TGG TGT T-3'*

Supplementary Table S2. List of primary antibodies

Protein	Primary antibody	Molecular weight	Isotype (source)	Used concentration	Cat #	Company
P-16	P16 (F-12)	16 kDa	Mouse IgG	1:1000	SC-1661	Santa Cruz
α-SMA	pAb to alpha smooth muscle Actin	30,35,37 kDa	Rabbit IgG	1:1000	Ab5694	Abcam
TGF-β	mAb to TGF beta1 (2Ar2)	50 kDa	Mouse IgG	1:1000	Ab64715	Abcam
FAP-α	pAb to fibroblast activation protein alpha	100,130 kDa	Rabbit IgG	1:1000	Ab53066	Abcam
SDF-1	pAb to SDF1	10-15	Rabbit IgG	1:1000	Ab9797	Abcam
DCN	Anti-hDecorin Antibody	37-45 kDa	Mouse IgG	1:500	MAB 143	R&D System
IL-6	Anti-interleukin-6 (IL-6) Antibody	21,26 kDa	Rabbit IgG	1:1000	701028	Invitrogen
Twist-1	Anti-Twist-1 antibody [10E4E6]	21 kDa	Mouse IgG	1:500	ab175430	Abcam
p-STAT-3	Phospho-STAT3(Y70) (D3A7) Antibody	79, 86 kDa	Rabbit IgG	1:1000	9145S	Cell Signaling
STAT-3	STAT-3 (124H6)	79, 86 kDa	Mouse IgG	1:1000	9139S	Cell Signaling
N-cadherin	N-cadherin Antibody (13A9)	110-140 kDa	Mouse IgG	1:500	14215S	Cell Signaling

E-cadherin	E-cadherin Antibody (4A2)	110-140 kDa	Mouse IgG	1:1000	14472s	Cell Signaling
Vimentin	mAb to vimentin (RV202) antibody	57,59 kDa	Mouse IgG	1:1000	Ab8978	Abcam
VEGF-A	Anti-VEGF antibody	45-52 kDa	Rabbit IgG	1:500	ab46154	Abcam
mTOR	mTOR (7C10) Antibody	250 kDa	Rabbit IgG	1:1000	2983S	Cell Signaling
pmTOR	P-mTOR (S2448) Antibody	250 kDa	Rabbit IgG	1:1000	2971S	Cell Signaling
AKT	AKT1 (C73H10) Antibody	50-60 kDa	Rabbit IgG	1:1000	2938S	Cell Signaling
p-AKT	p-AKT1 (T308) antibody	50-60 kDa	Rabbit IgG	1:1000	9275s	Cell Signaling
HIF-1 Alpha	HIF-1 Alpha antibody	120 kDa	Rabbit IgG	1:1000	3716S	Cell Signaling
IL-8	IL-8 (21-99) Antibody	26 kDa	Mouse IgG	1:1000	H0000357 6-M01	Abnova (Taipei, Taiwan)
AUF-1	Anti-AUF-1 Antibody	37-45 kDa	Rabbit IgG	1:1000	2605378	Millipore
P-JAK-2	P-JAK2 (Y1007/1008) Antibody	60-65 kDa	Rabbit IgG	1:1000	3771S	Cell Signaling

JAK-2	mAb to JAK2 (E132) Antibody	55-65 kDa	Rabbit IgG	1:1000	Ab32101	Abcam
p-NF_kB	p-NF_kB p65(Ser 536) Sc-33020	65 kDa	Rabbit IgG	1:1000	G1813	Santa Cruz
NF_kB	NF_kB p65(F-6) Sc-8008	65 kDa	Mouse IgG	1:1000	G2111	Santa Cruz
β-actin	Beta-Actin	42 kDa	Rabbit IgG	1:1000	4967L	Cell Signaling
CD-44	SAB 4300691 Anti-CD44 antibody	75-100 kDa	Rabbit IgG	1:1000	87152147 1	Sigma
CD-24	CD24 (SN3) sc-19585 Antibody	35-55 kDa	Mouse IgG	1:1000	B2117	Santa Cruz
ALDH-1	Anti-ALDH Antibody	50-52 kDa	Mouse IgG	1:1000	8178547	BD
EpCAM	EpCAM (D1B3) Antibody	40 kDa	Rabbit IgG	1:1000	2626S	Cell signaling
Snail	Snail (C15D3) Antibody	29 kDa	Mouse IgG	1:1000	3879	Cell signaling
GAPDH	GAPDH (14C10)	35,37 kDa	Rabbit IgG	1:1000	2118s	Cell Signaling

Supplementary Table S3. List of secondary antibodies

**Source of primary antibody**	**Secondary antibody**	**Used concentration**	**Cat #**	**Company**
Rabbit	Anti-rabbit IgG-HRP conjugate	1:10,000	(REF W4018)	Promega, WI, USA
Mouse	Anti-mouse IgG-HRP conjugate	1.5:10,000	(REF W4028)	

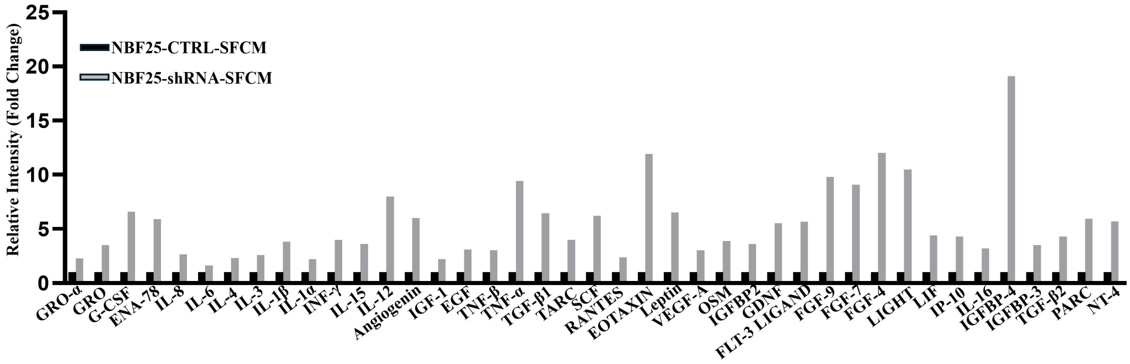

Supplementary Figure S1. Decorin downregulation in breast stromal fibroblasts enhances the secretion of several cytokines

SFCM from the NBF25-CTRL and NBF-25-shRNA cells were applied onto the human cytokine antibody array membrane (C5). The intensities of the spots in the NBF25-shRNA-SFCM cytokine array membrane were quantified by densitometric analysis and divided by the values obtained in the control for each protein and were presented as fold change.

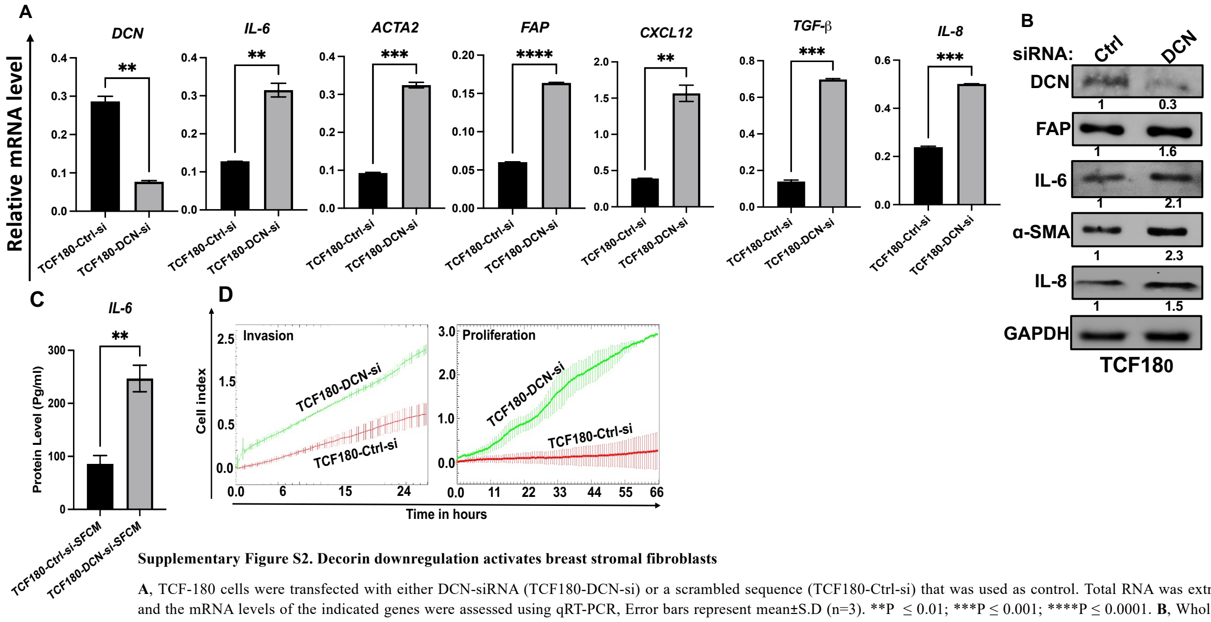

Supplementary Figure S2. Decorin downregulation activates breast stromal fibroblasts

A, TCF-180 cells were transfected with either DCN-siRNA (TCF180-DCN-si) or a scrambled sequence (TCF180-Ctrl-si) that was used as control. Total RNA was extracted and the mRNA levels of the indicated genes were assessed using qRT-PCR, Error bars represent mean±S.D (n=3). $^{**}P \leq 0.01$; $^{***}P \leq 0.001$; $^{****}P \leq 0.0001$. **B**, Whole-cell lysates were prepared from the indicated cells, and then were used for immunoblotting analysis using specific antibodies against the indicated proteins. **C**, SFCM from the indicated cells were collected after 24 h and the levels of the indicated protein were determined by ELISA. Error bars indicate mean±S.D (n=3). $^{**}P \leq 0.01$. **D**, exponentially growing cells were seeded, and cell invasion, and proliferation were assessed using the RTCA-DP xCELLigence System. Data are representative of different experiments performed in triplicate.

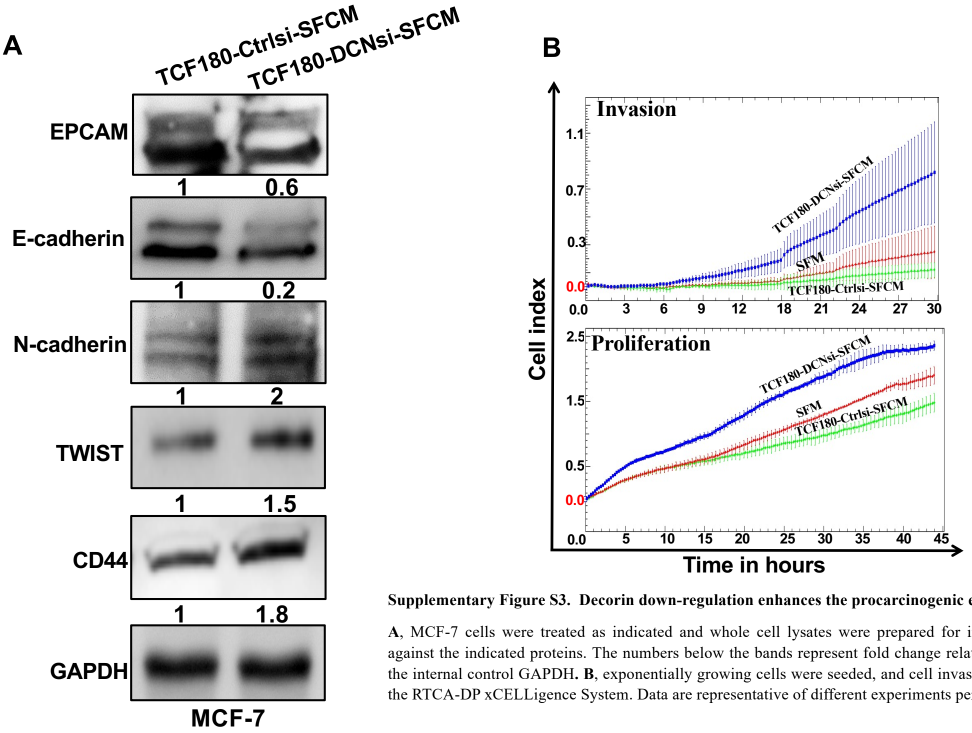

Supplementary Figure S3. Decorin down-regulation enhances the procarcinogenic effects of tumor counterpart fibroblasts

A, MCF-7 cells were treated as indicated and whole cell lysates were prepared for immunoblotting analysis using antibodies against the indicated proteins. The numbers below the bands represent fold change relative to the control after correction against the internal control GAPDH**. B**, exponentially growing cells were seeded, and cell invasion, and proliferation were assessed using the RTCA-DP xCELLigence System. Data are representative of different experiments performed in triplicate.
